# Metabolomic profiling of human lung tumor tissues – nucleotide metabolism as a candidate for therapeutic interventions and biomarkers

**DOI:** 10.1002/1878-0261.12369

**Published:** 2018-09-13

**Authors:** Paula Moreno, Carla Jiménez‐Jiménez, Martín Garrido‐Rodríguez, Mónica Calderón‐Santiago, Susana Molina, Maribel Lara‐Chica, Feliciano Priego‐Capote, Ángel Salvatierra, Eduardo Muñoz, Marco A. Calzado

**Affiliations:** ^1^ Instituto Maimónides de Investigación Biomédica de Córdoba (IMIBIC) Cordoba, Spain; ^2^ Unidad de Cirugía Torácica y Trasplante Pulmonar Hospital Universitario Reina Sofía Cordoba Spain; ^3^ Departamento de Biología Celular Fisiología e Inmunología Universidad de Córdoba Cordoba, Spain; ^4^ Innohealth Group Madrid Spain; ^5^ Departamento de Química Analítica Universidad de Córdoba Cordoba, Spain

**Keywords:** biomarkers, lung cancer, metabolomics, nucleotides

## Abstract

Although metabolomics has attracted considerable attention in the field of lung cancer (LC) detection and management, only a very limited number of works have applied it to tissues. As such, the aim of this study was the thorough analysis of metabolic profiles of relevant LC tissues, including the most important histological subtypes (adenocarcinoma and squamous cell lung carcinoma). Mass spectrometry‐based metabolomics, along with genetic expression and histological analyses, were performed as part of this study, the widest to date, to identify metabolic alterations in tumors of the most relevant histological subtypes in lung. A total of 136 lung tissue samples were analyzed and 851 metabolites were identified through metabolomic analysis. Our data show the existence of a clear metabolic alteration not only between tumor vs. nonmalignant tissue in each patient, but also inherently intrinsic changes in both AC and SCC. Significant changes were observed in the most relevant biochemical pathways, and nucleotide metabolism showed an important number of metabolites with high predictive capability values. The present study provides a detailed analysis of the metabolomic changes taking place in relevant biochemical pathways of the most important histological subtypes of LC, which can be used as biomarkers and also to identify novel targets.

**Abbreviations**
AC: adenocarcinoma
ADSL: adenylosuccinate lyase
ATIC: 5‐aminoimidazole‐4‐carboxamide ribonucleotide formyltransferase/IMP cyclohydrolase
LC: lung cancer
NSCLC: non‐small‐cell lung cancer
PCA: principal component analysis
PLS‐DA: partial least squares discriminant analysis
QA: quality assurance
QC: quality control
SAH: *S*‐adenosylhomocysteine
SCC: squamous cell lung carcinoma
TGCA: The Cancer Genome Atlas
UPLC/MS/MS: ultra‐performance liquid chromatography/tandem mass spectrometry

## 1 Introduction

Nowadays, lung cancer (LC) is the leading cause of death from cancer in the world. The most common type of LC is non‐small‐cell LC (NSCLC), which represents 85% of all LCs. The most important histological subtypes include adenocarcinoma (AC), squamous cell lung carcinoma (SCC) and large‐cell carcinoma, of which AC and SCC represent ~ 90% of all cases (Falco *et al*., 2016). Generally, diagnosis takes place after the first symptoms have already appeared, which normally happens at an advanced stage. Although different methods such as computerized tomography scans and bronchoscopy have contributed to detection of the disease, they do not increase the likelihood of early diagnosis (National Lung Screening Trial Research Team *et al*., 2011).

In the search for new cancer markers to be applied with a minimally invasive screening methodology, the field of metabolomics has enjoyed considerable interest in the last years (Aboud and Weiss, 2013). Samples that can be analyzed in metabolomics include cells, fluids or tissues, biofluids such as urine, total blood, plasma, serum and saliva being the most widely used because of the minimally invasive methods used to obtained them. Although there is an increasing interest in studies using tumor tissues, few analyses have been carried out, mainly due to the difficulty of obtaining this type of sample (Armitage and Southam, 2016).

The analysis of the metabolomics profile characterizing LC is still in its early stages. Nevertheless, different approximations have been developed to identify altered metabolites in LC through monitoring several types of samples such as serum (Kumar *et al*., 2017), plasma (Louis *et al*., 2017), urine (Haznadar *et al*., 2016), sputum (O'Shea *et al*., 2016) or sweat (Calderon‐Santiago *et al*., 2015). In contrast, to date only a very limited number of works have directly analyzed metabolic differences in tissues. The first approach was performed by Fan *et al*. (2009), who analyzed ^13^C‐glucose‐labeled tissue extracts from 12 patients to show an alteration in the Krebs cycle activity and the pyruvate carboxylation. Later, Rocha *et al*. (2010) analyzed the differences between tumor and non‐involved adjacent lung tissues of 12 samples and proved a clear increase in phosphocholine, lactate and glycerophosphocholine levels in tumors, whereas there was a decrease in inosine/adenosine, acetate, myo‐inositol and glucose. That same year, Jordan *et al*. (2010) described the analysis of tissue samples from 14 subjects with AC (nine subjects) and SCC (five subjects) compared with control samples (seven subjects). In 2011, Hori *et al*. (2011) performed a metabolomic analysis of LC in seven patients, finding significant changes in 40 metabolites. Kami *et al*. (2013) reported a metabolomic profiling of nine samples of lung tumor tissues, proving a high glycolytic activity in them. Finally, the S. Miyamoto group has recently described different metabolic perturbations associated with AC (Fahrmann *et al*., 2017; Wikoff *et al*., 2015) and Rocha *et al*. (2015) have utilized ^1^H NMR metabolomics to analyze matched tumors and adjacent control tissues from 56 subjects with different types of primary lung carcinomas.

Although the use of monitoring metabolic levels as a tool to detect the early stages in some oncological diseases can be applied to easy‐to‐obtain samples, such as serum or plasma, knowing the changes originally experienced by the tissue is especially important in the search for these markers. This is why the aim of this study has been the thorough analysis of the metabolic profile of a relevant number of LC tissues belonging to the most prevalent histological subtypes (AC and SCC) and their corresponding adjacent healthy tissue from the same patient. As far as we are aware, this study is to date the widest in terms of the number of samples and histological subtypes analyzed. The obtained data prove the presence of biochemical changes at tissue level in the different LC samples, which can be useful for identifying biomarkers and to increase the knowledge of the metabolic reprogramming developed during tumorigenesis.

## 2 Materials and methods

### 2.1 Patient cohorts

Lung tissue samples from patients treated surgically for primary LC were obtained from University Hospital Reina Sofia (Cordoba, Spain). This research study was conducted in accordance with the Helsinki Declaration and was approved by the Cordoba Clinical Research Ethics Committee. Tissue samples were stored and managed by the Cordoba node belonging to the Biobank of the Andalusian Health Service (Servicio Andaluz de Salud – SAS). All patients signed a written informed consent document indicating their voluntary donation. All samples were histologically reviewed and classified, frozen and stored at −80 °C until analysis, and the remaining specimen was formalin‐fixed and paraffin‐embedded for conventional diagnostic studies and immunohistochemical analysis. None of the patients received chemotherapy or radiation therapy before the operation. Clinicopathological data were prospectively collected.

### 2.2 Metabolomic analysis

#### 2.2.1 Sample preparation

Metabolomic profiling analysis was performed by Metabolon as previously described (Reitman *et al*., 2011). Samples were prepared using the automated microlab star® system from Hamilton Company (Reno, NV, USA). A recovery standard was added prior to the first step in the extraction process for quality control purposes. Samples were prepared using an aqueous methanol extraction process to remove the protein fraction while allowing maximum recovery of small molecules. The resulting extract was divided into four fractions: one for analysis by ultra‐performance liquid chromatography/tandem mass spectrometry (UPLC/MS/MS; positive mode), one for UPLC/MS/MS (negative mode), one for GC/MS, and one for backup. Samples were placed briefly on a TurboVap® (Zymark, Hopkinton, MA, USA) to remove the organic solvent. Each sample was then frozen and dried under vacuum. Samples were then prepared for the appropriate instrument, either UPLC/MS/MS or GC/MS.

#### 2.2.2 UPLC/MS/MS

The LC/MS portion of the platform was based on Waters ACQUITY ultra‐performance liquid chromatography and a Thermo‐Finnigan linear trap quadrupole mass spectrometer, which consisted of an electrospray ionization source and a linear ion‐trap mass analyzer (Evans *et al*., 2009). The sample extract was dried and then reconstituted in acidic or basic LC‐compatible solvents, each of which contained 11 or more injection standards at fixed concentrations to ensure injection and chromatographic consistency. One aliquot was analyzed using acidic positive ion optimized conditions and the other using basic negative ion optimized conditions in two independent injections using separate dedicated columns. Extracts reconstituted in acidic conditions were gradient‐eluted using water and methanol containing 0.1% formic acid, and the basic extracts, which also used water/methanol, contained 6.5 mm ammonium bicarbonate. The MS analysis alternated between MS and data‐dependent MS^2^ scans using dynamic exclusion. Raw data files are archived and extracted as described below.

#### 2.2.3 GC/MS

The samples destined for GC/MS analysis were re‐dried under vacuum desiccation for a minimum of 24 h prior to being derivatized under dried nitrogen using bis‐trimethylsilyl‐trifluoroacetamide. The GC column was 5% phenyl and the temperature ramp was from 40 to 300 °C in a 16‐min period. Samples were analyzed on a Thermo‐Finnigan Trace DSQ fast‐scanning single‐quadrupole mass spectrometer using electron impact ionization. The instrument was tuned and calibrated for mass resolution and mass accuracy on a daily basis. The information output from the raw data files was automatically extracted as discussed below.

#### 2.2.4 Quality assurance (QA)/quality control (QC)

For QA/QC purposes, additional samples were included with each day's analysis. These samples included extracts of a pool of well characterized human plasma, extracts of a pool created from a small aliquot of the experimental samples and process blanks. QC samples were spaced evenly among the injections and all experimental samples were randomly distributed throughout the run. A selection of QC compounds was added to every sample for chromatographic alignment, including those being tested. These compounds were carefully chosen so as not to interfere with the measurement of the endogenous compounds.

#### 2.2.5 Data extraction and compound identification

Raw data were extracted, peak‐identified and QC‐processed. Compounds were identified by comparison with library entries of purified standards or recurrent unknown entities. Biochemical identifications are based on three criteria: retention index within a narrow window of the proposed identification, nominal mass match to the library ± 0.2 amu and the MS/MS forward and reverse scores between the experimental data and authentic standards. The MS/MS scores are based on a comparison of the ions present in the experimental spectrum with the ions present in the library spectrum. Although there may be similarities between these molecules based on one of these factors, all three data points can be utilized to distinguish and differentiate biochemicals. Based on the literature and on KEGG/HMDB databases, metabolites were annotated to one of eight ‘superpathways’ corresponding to their general metabolic processes (amino acid, lipid, carbohydrate, nucleotide, peptide, energy, cofactors and vitamins, and xenobiotics), and to one of 73 ‘subpathways’ representing more specific metabolic pathways or biochemical subclasses. The three networks (metabolite, subpathway and superpathway) together depict the hierarchical map and have been used in previous studies (Krumsiek *et al*., 2012; Poisson *et al*., 2015) (Data S1).

#### 2.2.6 Data analysis and statistics

A final.csv containing information about the area of metabolites in all samples was imported in r (URL http://www.R-project.org) through the interface of rstudio (URL https://www.rstudio.com) for further statistical analysis and graphics plot. Missing values (if any) are assumed to be below the level of detection. However, metabolites detected in all samples from one or more groups but not in samples from other groups were assumed to be near the lower limit of detection in the groups in which they were not detected. In this case, the lowest detected level of these metabolites was imputed for samples in which that metabolite was not detected. Pathways were assigned for each metabolite, allowing examination of overrepresented pathways. After imputation with minimum observed values, the datasets were log‐transformed for further analyses. Principal component analysis (PCA) and partial least squares discriminant analysis (PLS‐DA) were used respectively as unsupervised and supervised multivariate approaches to visualize metabolic changes occurring in tumor tissues as compared with normal tissue. The PLS‐DA was applied with a 10‐fold internal validation. A paired two‐sample *t*‐test was used to identify metabolites that differed significantly between the tumor and normal tissue. A Welch two‐sample *t*‐test was applied on the log‐transformed tumor/normal tissue ratios to compare metabolite levels across different tumor types. The same analysis was applied at superpathway level. The r packages employed for statistical analysis and plot generation were pheatmap 1.0.8, ggplot2 2.2.1, mixOmics 6.3.1 and MetaboAnalyztR 0.0.0.9.

### 2.3 Histological analysis

Immunohistochemical staining was performed on formalin‐fixed, paraffin‐embedded samples. Sections of 5 μm were deparaffinized in xylene and rehydrated in a graded ethanol series. Staining with the rabbit polyclonal 5‐aminoimidazole‐4‐carboxamide ribonucleotide formyltransferase/IMP cyclohydrolase (ATIC) antibody (HPA021012; Sigma‐Aldrich, St. Louis, MO, USA) at 1 : 200 dilution or anti‐adenylosuccinate lyase (ADSL) antibody (HPA000525; Sigma‐Aldrich) at 1 : 100 dilution was performed overnight at 4 °C. Staining was evaluated as follows: 0, no staining or faint staining in < 10% of the cells; 1,10–25% of stained cells; 2, 26–50% of stained cells; 3, 51–75% of stained cells; 4, more than 74% of stained cells.

### 2.4 mRNA extraction and qPCR

Total RNA was isolated from tissues stored in RNAlater® using the RNeasy Plus Universal Mini Kit, the TissueLyser LT and a QIAcube (Qiagen, Hilden, Germany) according to the manufacturer's protocol. Total RNA concentration and integrity (RNA integrity number (RIN) > 9) were analyzed using an Experion® automated electrophoresis station (Bio‐Rad, Hercules, CA, USA). Isolated RNA was converted to cDNA using the iScript cDNA Synthesis Kit (Bio‐Rad). Real‐time PCR was employed with GoTaq qPCR Master Mix (Promega, Madison, WI, USA) in an iCYCLER detection system (Bio‐Rad). The amplification profile consisted of an initial denaturation for 5 min at 95 °C and then 39 cycles of 30 s at 95 °C, annealing for 10 s at a temperature of 60 °C (*GMPR*,* GMPS, IMPDH2, XDH, ADA* genes); 57.3 °C (*ADSL, ADSS, ATIC* genes); 63 °C (*HPRT* gene) or 62.2 °C (*ITPA* gene), 30 s at 72 °C for extension and one denaturation step of 1 min at 97 °C. Amplification efficiencies were validated and normalized against β‐actin, and fold change in genetic expression was calculated using the 2^−ΔΔCt^ method. The following primers were used: *ADA*, forward, 5′‐CCATTTCTGCACACACGTATACC‐3′, reverse, 5′‐TGGCCAGGGCACATAATCA‐ 3′; *IMPDH2*, forward, 5′‐AGGGAAAGTTGCCCATTGTAAA‐3′, reverse, 5′‐TGGGTAGTCCCGATTCTTCTTC‐3′; *GMPS*, forward, 5′‐ATGGCTCTGTGCAACGGAG‐3′, 5′‐CCTCACTCTTCGGTCTATGACT‐3′; *GMPR*, forward, 5′‐AATGTAGCCGTGAGTTCAGGC‐3′, 5′‐GCCATAATGGTGTGTTCAGGAAA‐3′; *XDH*, forward, 5′‐ACCGCTTCCACTACTTCAGCTAT‐3′,5′‐TTAGACTGGAGCCAACATCCATG‐3′; *ATIC*, forward, 5′‐TCTGATGCCTTCTTCCCTTT‐3′, 5′‐AGGTTCGTATGAGCGAGGAT‐3′; *HPRT1*, forward, 5′‐AATTATGGACAGGACTGAACGTCTTGCT‐3′, 5′‐TCCAGCAGGTCAGCAAAGAATTTATAGC‐3′; *ITPA*, forward, 5′‐AGCTGGCTCTGCTCTGAGAAA‐3′, 5′‐GCTGTAGGAGAGAGCAGTGAATCC‐3′; *ADSL*, forward, 5′‐TGGTGACAGAAAAGGCAGGA‐3′, 5′‐GCGTATGTCGGTGCAAATCT‐3′; *ADSS*, forward, 5′‐ AGGGGTAGAGAGTTTGGTGT‐3′, 5′‐GTGCCAACGCAGTAAATCCA‐3′.

### 2.5 Analysis of public data from cancer genomics studies

To analyze *ADSL* and *ATIC* genetic alterations including mRNA expression *z*‐scores (Microarray, threshold 2.0), data from The Cancer Genome Atlas Research Network (TCGA; Lung AC and Lung Squamous Cell Carcinoma Provisional sequenced tumors sample sets) were analyzed using cbioportal software ( http://www.cbioportal.org/) and visualized using the standard Oncoprint output showing altered columns. The effect of *ADSL* and *ATIC* genetic expression on LC patient prognoses was evaluated by Kaplan–Meier survival curves of LC patients with low or high expression of *ADSL* and *ATIC* with data from Kaplan–Meier plotter (kmplotter; http://www.kmplot.com/analysis). Data were collected using all available patients restricted to histological subtype for 120 months. Kaplan–Meier plots were constructed using graphpad prism version 6.0c (GraphPad Software, La Jolla, CA, USA), and a log‐rank test was calculated to determine differences in overall survival according to *ADSL* and *ATIC* mRNA levels using spss 11.5.0 for Windows (IBM Corp., Armonk, NY, USA).

## 3 Results

### 3.1 Metabolomic profile of lung tissues

A total of 136 lung tissue samples were selected, and histologically reviewed and classified as AC (*n* = 33) or SCC (*n* = 35), together with their corresponding samples of adjacent normal lung tissue from the same patient. All the samples were obtained, classified and stored by pathologists following biobank quality assurance steps. The clinicopathological characteristics of the entire patient cohort are summarized in Table 1. A total of 851 metabolites were identified through metabolomic analysis in both histological subtypes, which gave a comprehensive snapshot of the metabolic state of lung tissues. Unsupervised analysis by PCA applied to the complete dataset revealed a clear discrimination pattern for both histological subtypes, AC and SCC, vs. normal lung tissue, as shown in Fig.  S1. This discrimination was supported on critical metabolic changes occurring in lung tissues. In fact, we identified a total of 280 compounds in AC and 623 in SCC with significant concentration differences between the experimental groups, and a total of 237 metabolites common to both (Data S1).

**Table 1:** Patient characteristics

Characteristics	AC (*n* = 33)	Squamous cell (*n* = 35)	*P*‐value
Age (mean)	62.11 ± 9.73	68.71 ± 7.46	0.002
Sex			
Male	24	35	0.032
Female	9	0	
Comorbidities	26	33	0.018
Neoplasms	8	2	0.54
Metastases in follow‐up	3	2	0.005
Tumor size	3.52 ± 1.98	4.5 ± 2.01	0.039
SUV(max)*	11.07 ± 8.19	13.4 ± 5.73	0.15
pTNM			
IA	10	5	0.38
IB	7	12	
IIA	6	7	
IIB	4	6	
IIIA	5	5	
IIIB	1	0	
Grade differentiation			
I	4	2	0.11
II	16	16	
III	11	6	
N.S.	2	11	

Data available in 50 cases.

**Figure 1:**
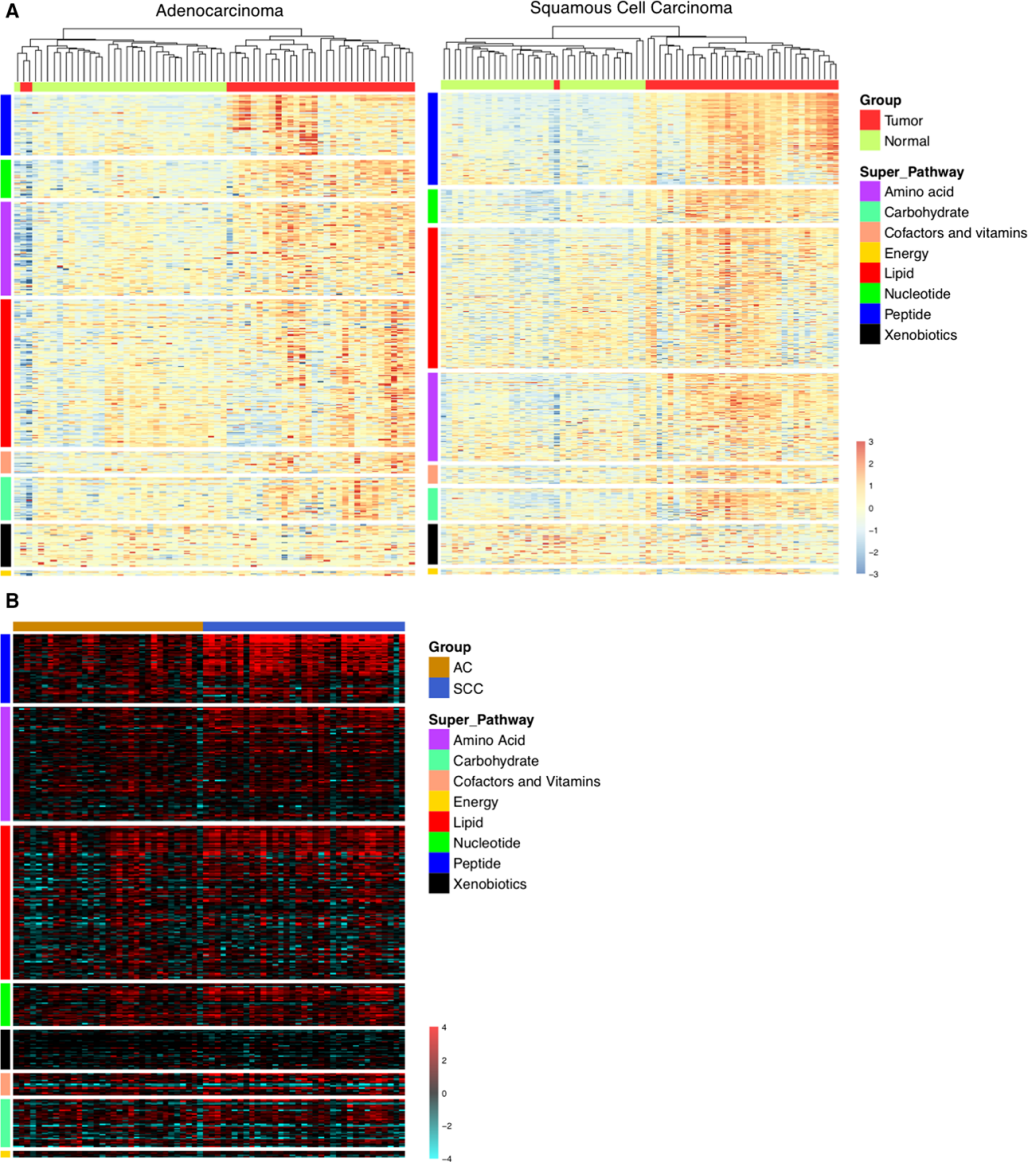
Heatmap representation of the metabolite levels obtained from each metabolomic assay. (A) The values are scaled using the *Z*‐score. The *Y*‐axis order (metabolites) reflects the statistical difference between metabolite levels in tumor and normal tissues at superpathway and metabolite levels. The *X*‐axis order (sample) is set after applying a hierarchical clustering algorithm, using unweighted pair group method with arithmetic mean (UPGMA) as the method and a Pearson's correlation of 1 as the metric. (B) The log‐transformed tumor/normal tissue ratios (logFC) for both carcinomas are ordered according to the statistical difference between metabolite level changes at superpathway and metabolite levels.

According to these results, we analyzed the different metabolic profiles obtained from normal and tumor tissues through visualization in heatmaps, where data were normalized and prioritized in different metabolic pathways, and also ordered according to the differential expressions between tumor and healthy tissues. As shown in Fig. 1A, the obtained results divide the tumor and normal tissue samples into two clearly different groups in nearly all cases. Although there are differences between AC and SCC in the hierarchy of metabolic pathways, the most altered in tumor tissue are those related to peptides, nucleotides, amino acids and lipids. Next, we examined the expression changes found between the same patient samples (tumor vs. healthy) in the list of common metabolites of both pathologies. As shown in Fig. 1B, although major differences were not detected, there was an increase in the induction levels of metabolites related to peptides and amino acids in the case of SCC. Together, these data show the existence of a clear metabolic alteration not only between tumor vs. healthy tissue in each patient, but also inherently intrinsic to both pathologies.

### 3.2 Metabolism of glucose in lung cancer

**Figure 2:**
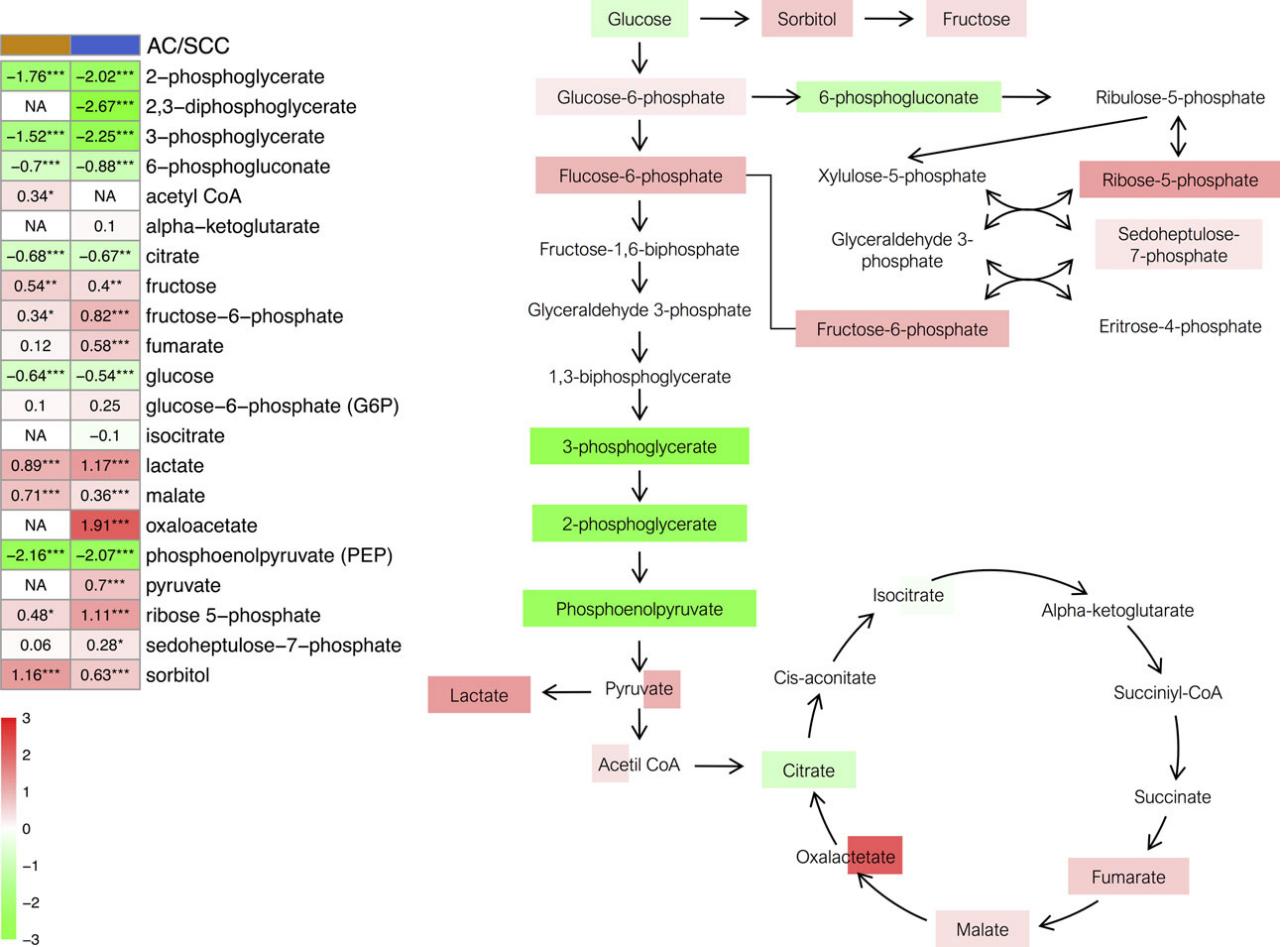
Glucose metabolism pathway. The heatmap shows the mean of the log‐transformed tumor/normal ratios for metabolites in AC (orange) and squamous cell cancer (blue). Increase (red) or decrease (green) in metabolite level. *P < 0.05, **P < 0.01 and ***P < 0.001.

We next performed a detailed analysis of relevant metabolites showing significant changes hierarchically in the most important pathways. As shown in Fig. 2, we detected a significant decrease in the levels of various glycolysis metabolites involved in both tumor subtypes, such as glucose, 3‐ and 2‐phosphoglycerate and phosphoenolpyruvate. These were also accompanied by a significant accumulation of lactate and pyruvate (at least in SCC), the final products of glycolysis. A significant increase in the pentose phosphate pathway was also evident, including ribose, ribose 5‐phosphate, sorbitol and fructose. As regards the tricarboxylic acid pathway metabolites, we observed an increase in several intermediate metabolites, such as fumarate and malate in both tumor subtypes, and oxalacetate in SCC. In the same sense, we found other changes suggesting an increase in the use of glucose; for example, a significant increase in the levels of sugar alcohols and amino sugars.

### 3.3 Glutathione levels, oxidative stress markers and polyamine metabolism

**Figure 3:**
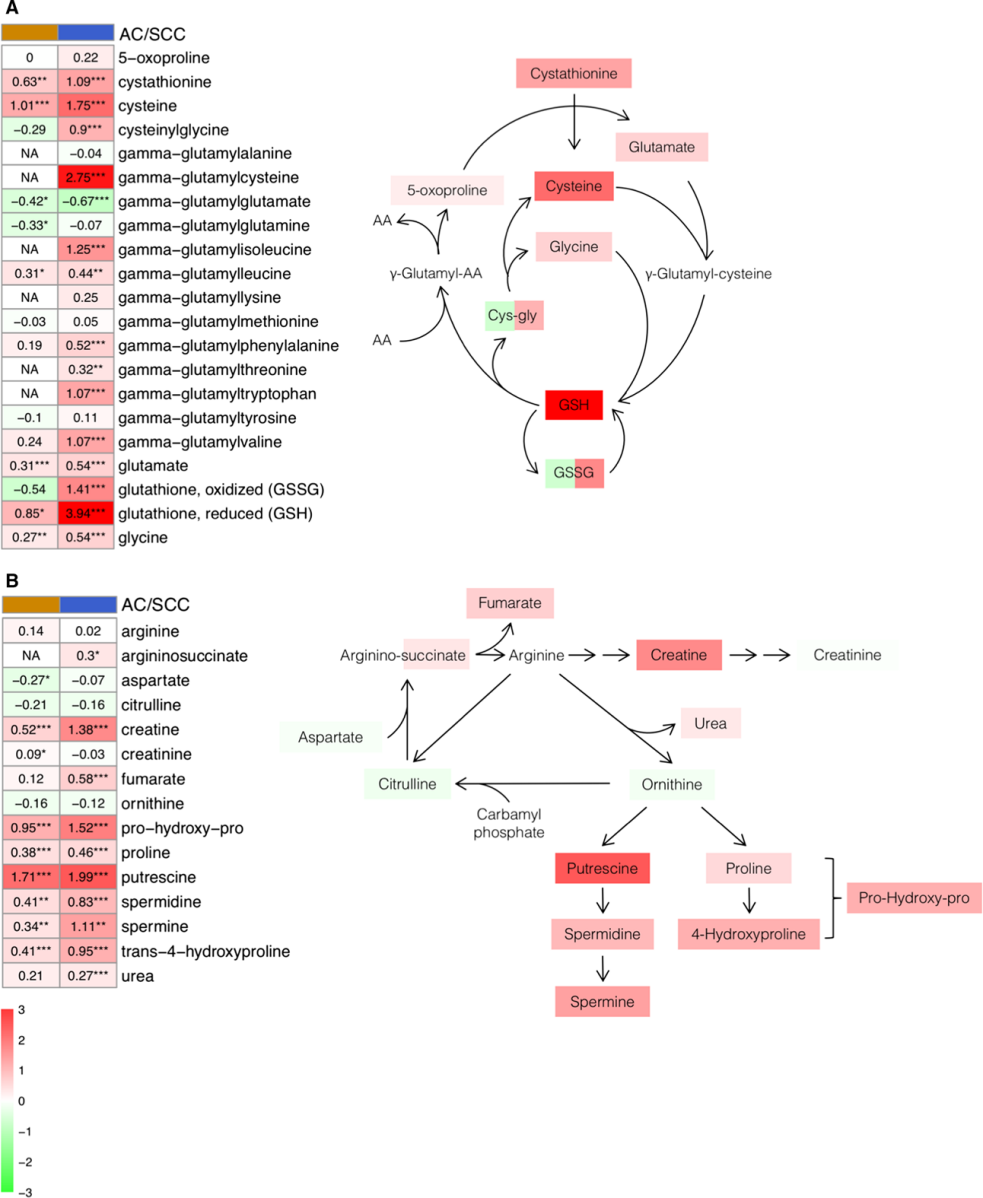
Glutathione (A) and polyamines (B) metabolism pathways. Heatmaps show the mean of the log‐transformed tumor/normal ratios for metabolites in AC (orange) and squamous cell cancer (blue). Increase (red) or decrease (green) in metabolite level. *P < 0.05, **P < 0.01 and ***P < 0.001.

Glutathione plays a determining role in antioxidant defense, redox homeostasis and toxin detoxification (Jozefczak *et al*., 2012). The obtained results show an increase in methionine sulfoxide (MetO) levels in both tumor subtypes compared with healthy tissue, along with reduced glutathione. Similarly, we observed an increase in cystathionine, cysteine and glycine, which are limiting components of glutathione synthesis (Fig. 3A). In contrast, there are differences between AC and SCC in the changes of both oxidized glutathione levels and cysteine‐glycine levels. Changes detected in gamma‐glutamyl amino acid levels suggest an alteration in the levels of glutathione. Equally, tumor samples showed high levels of antioxidants, such as ascorbate and alpha/gamma‐tocopherol. These findings show an accumulation of oxidative stress markers and antioxidants.

Arginine metabolism has an important role in the remodeling of the extracellular matrix and the production of polyamines, creatine and nitric oxide (Rath *et al*., 2014). As shown in Fig. 3B, we observed a light increase in arginine, ornithine and citrulline levels, as well as in aspartate in the tumor tissue compared with the healthy tissue. In contrast, tumor tissues showed significant high levels of polyamines such as putrescine, spermine and spermidine, as well as numerous molecules related to polyamines, such as *N*‐acetylputrescine and 5‐methylthioadenosine (MTA). As regards the remodeling markers of the extracellular matrix, we found a significant increase in proline, trans‐4‐hydroxyproline and pro‐hydroxy‐pro, as well as in asymmetric dimethylarginine.

### 3.4 Fatty acids, lipid mediators and one‐carbon metabolism

**Figure 4:**
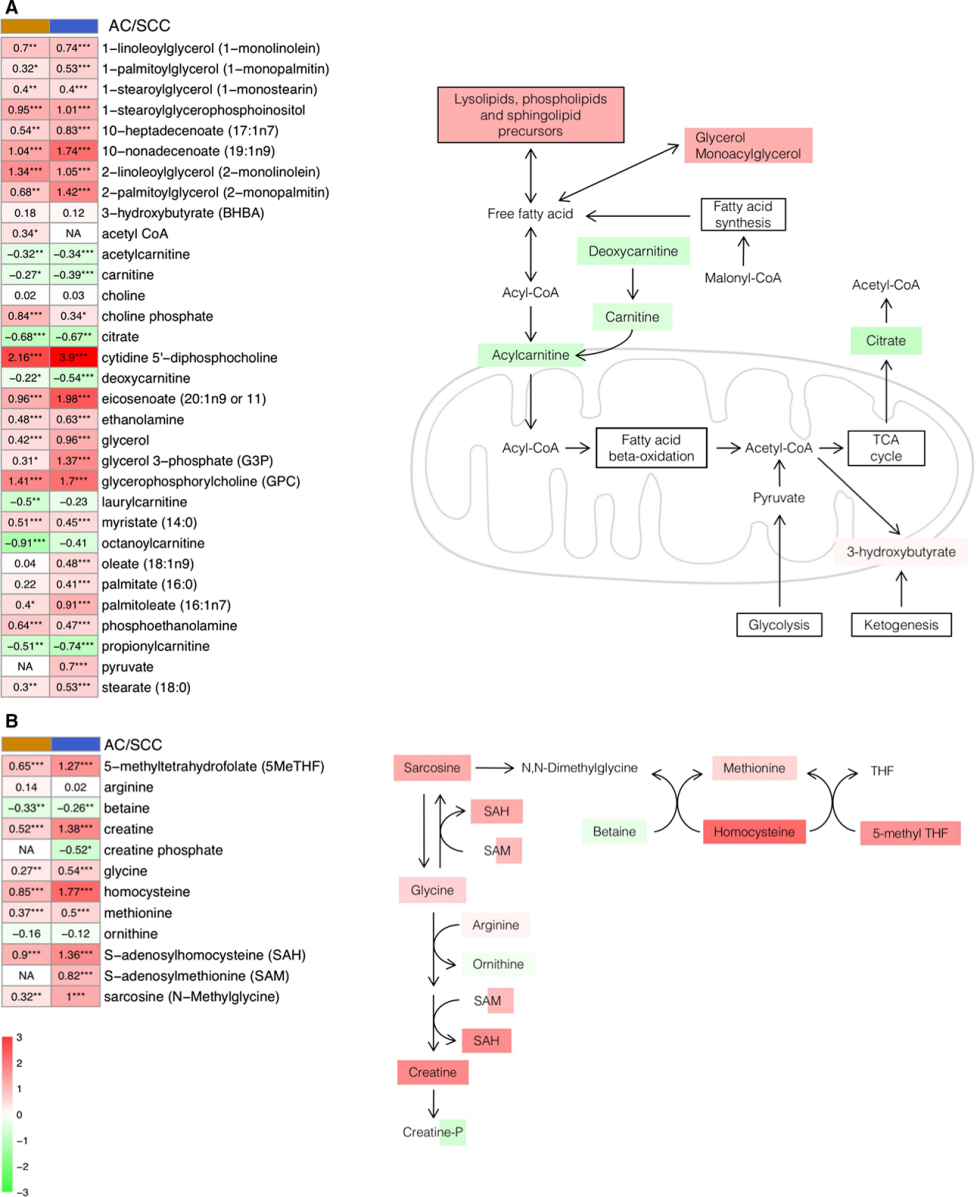
Fatty acids (A) and carbon (B) metabolism pathways. Heatmaps show the mean of the log‐transformed tumor/normal ratios for metabolites in AC (orange) and squamous cell cancer (blue). Increase (red) or decrease (green) in metabolite level. *P‐value < 0.05, **P‐value < 0.01 and ***P‐value < 0.001.

Fatty acids are a valuable energy source for mitochondrial oxidation and ATP cell generation (Lehner and Quiroga, 2016). The metabolomic profile of tumor tissues showed a significant increase in monoacylglycerols and glycerol, as well as elevated levels of medium‐ and long‐chain fatty acids (Fig. 4A). Additionally, multiple phospholipid metabolites, including choline phosphate, phosphoethanolamine, cytidine 5′‐diphosphocholine, glycerophosphorylcholine and glycerol 3‐phosphate, were significantly elevated. We observed a significant increase in the levels of essential fatty acids such as dihomo‐linolenate and arachidonate. In contrast, citrate, carnitine and carnitine conjugated lipids were significantly lower. Thus, these observations suggest lipid oxidation may be disrupted in lung tumor tissue.

Some previous studies have associated carbon metabolism with carcinogenesis, mainly due to its role in DNA synthesis (Newman and Maddocks, 2017). As shown in Fig. 4B, we detected a significant increase in the levels of glycine, sarcosine, *S*‐adenosylhomocysteine (SAH), methionine and homocysteine. By contrast, a decrease in the levels of betaine and ornithine was observed. These changes were also accompanied by an increase in the levels of 5‐methyltetrahydrofolate, as well as in the number of metabolites related to riboflavin metabolism in both tumor subtypes.

### 3.5 Nucleotide catabolism

**Figure 5:**
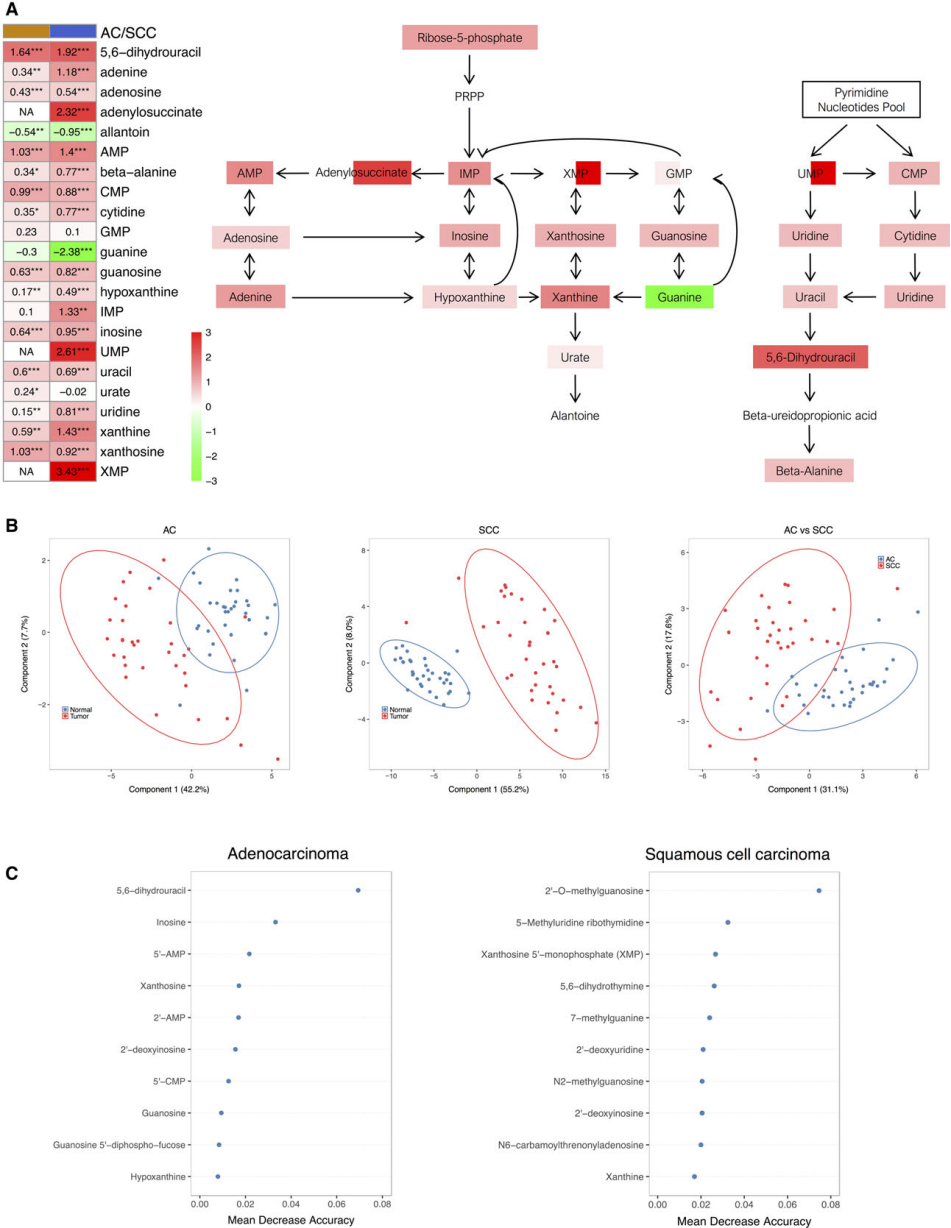
(A) Nucleotide metabolism pathway. The heatmap shows the mean of the log‐transformed tumor/normal ratios for metabolites in AC (orange) and squamous cell cancer (blue). Increase (red) or decrease (green) in metabolite level. *P < 0.05, **P < 0.01 and ***P < 0.001. (B) PLS‐DA obtained for AC and SCC tissues using significant nucleotides and metabolites between normal and cancerous tissue. The datasets included those metabolites that were statistically significant in AC and SCC compared with normal tissue (37 and 61 metabolites for AC and SCC, respectively). The third PLS‐DA was obtained using the fold change value of each metabolite between normal and cancerous tissue in order to compare both types of carcinomas. For this purpose, we only considered those metabolites that were commonly altered in AC and SCC cases compared with normal tissue (34 metabolites). (C) Top 10 nucleotides and derivatives ranked by their discriminatory capability (expressed as mean decrease accuracy) provided by random forest analysis for AC and SCC cases.

As explained above in Fig. 2, we observed a significant increase in ribose‐5‐phosphate levels in tumor tissue, the initial substrate in purine pathway. A detailed analysis of this pathway also shows a significant increase in its first products, such as AMP or IMP; Fig. 5A). In addition, we detected a significant accumulation of multiple products from purine catabolism such as inosine, hypoxanthine, xanthine and xanthosine, as well as adenosine, adenine and guanosine. In contrast, it should be noted that we found significant changes in the expression of xanthosine 5′‐monophosphate only in SCC, as well as a decrease in guanine levels. We also detected light changes in urate expression, especially in AC, as a result of the degradation of purine metabolism. Similarly, alterations in the pyrimidine pathway were detected. Significantly high levels of CMP were detected in tumor tissue, as well as pyrimidine metabolites such as uridine and cytidine, which were accompanied by significantly elevated levels of the catabolites uracil, dihydrouracil and beta‐alanine.

### 3.6 Analysis of the genetic expression profile in purine metabolism: histological expression of ATIC and ADSL

As we have shown above in Fig. 1A, the most altered metabolic pathways in both histological subtypes were those related to peptides and nucleotides. This statistical difference between metabolite levels in tumor and normal tissues, together with the clear relevance of the nucleotide metabolism in the development of tumorigenesis, led us to analyze this pathway in more detail. The first evaluation consisted in an unsupervised analysis by PCA to look for discrimination patterns using metabolites included in the nucleotide metabolic pathway for AC and SCC vs. normal tissue (Fig.  S2). The clearest discrimination in the metabolism of nucleotides was the one found between SCC and normal tissues. Nevertheless, discrimination trends were also observed between AC and normal tissues, and between AC and SCC tissues.

The same scenario was found by PLS‐DA (Fig. 5B), which showed a clear discrimination effect both in AC and SCC vs. normal tissue when considering altered metabolites. The percentage of variability explained was above 50% in both cases. An additional analysis considering only those metabolites commonly altered in AC and SCC was performed to study the existence of discrimination patterns, showing the existence of differences in the levels of metabolites. Accuracy, *R* ^2^ and *Q* ^2^ parameters for the three PLS‐DA models, detailed in Fig.  S3, provide an overview of their discriminatory performance.

**Figure 6:**
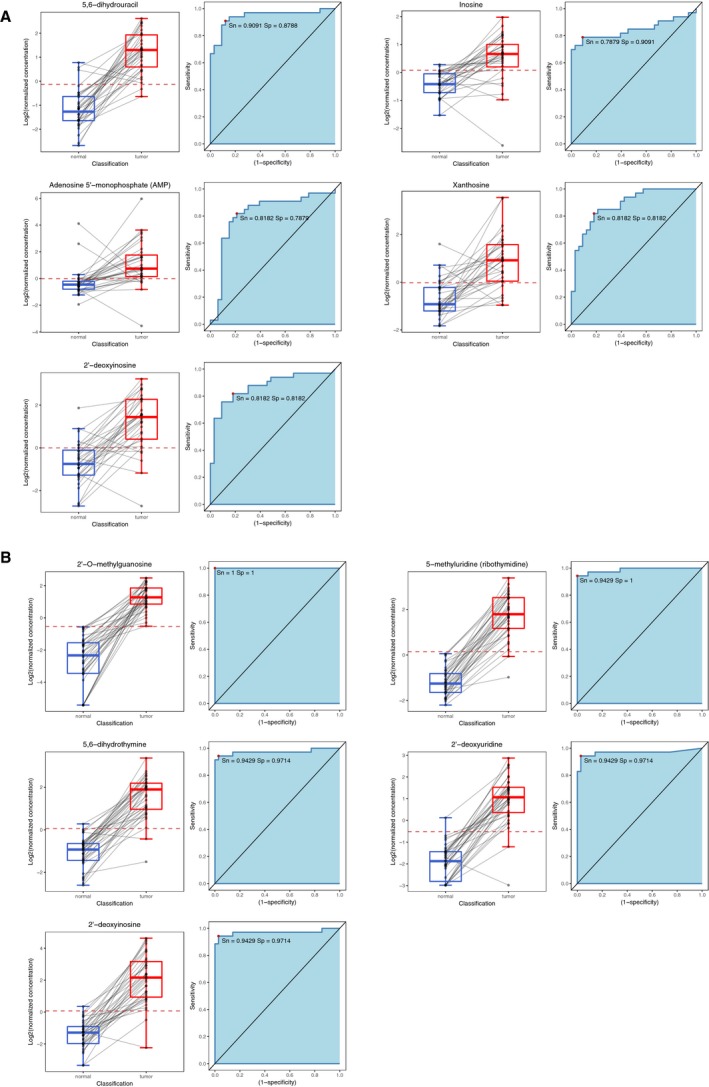
Box plots and receiver operating characteristic curves obtained for the top five nucleotides and metabolites to discriminate between normal and cancerous tissue according to the random forest analysis for AC (A) and SCC (B). The area under the curve is also shown, as well as the sensitivity and specificity for the threshold providing highest accuracy.

Further random forest analysis provided the top 10 metabolites with the highest discrimination power for AC and SCC tissues vs. the corresponding normal tissues (Fig. 5C). 5,6‐Dihydrouracil and inosine were the two metabolites with the highest discriminatory capability for AC, and 2′‐O‐methylguanosine and 5‐methyluridine for SCC cases. We analyzed the predictive capability of the top five metabolites for diagnostic purposes. Figure 6 shows the box plots and receiver operating characteristic curves obtained for each compound for AC and SCC cases.

**Figure 7:**
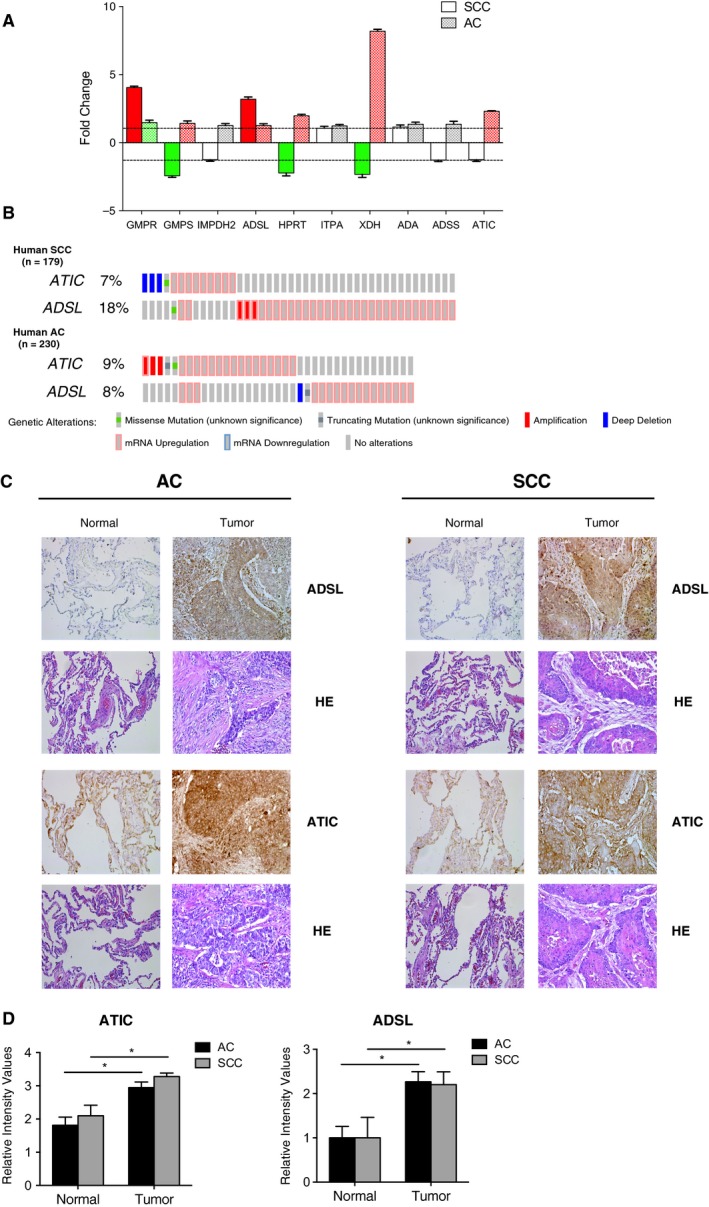
Purine metabolism and expression of ATIC and ADSL. (A) Total RNA was extracted from AC and SCC tissues, integrity evaluated and changes in expression of the indicated genes in tumor samples compared with normal lung samples from the same patient analyzed by qPCR and expressed as a fold change. Amplification efficiencies were validated and normalized against β‐actin, and fold change in genetic expression was calculated using the 2^−ΔΔCt^ method. Results are given as mean ± SD. (B) Genetic alterations in *ADSL* and *ATIC* genes in AC and SCC human samples. Data from TCGA were analyzed using cbioportal software. Each column represents a patient and displays only the percentage of altered cases. (C) Expression of ADSL and ATIC in AC and SCC analyzed by immunohistochemistry. Representative images of lung tumor and adjacent normal tissue stained with ADSL or ATIC antibody (×20) and hematoxylin–eosin (HE; ×20). (D) ATIC and ADSL expression in AC and SCC compared with surrounding healthy tissue. Expression was quantified as detailed in Materials and methods. The results are expressed as relative intensity values and represent the mean ± SD. *P < 0.05.

**Figure 8:**
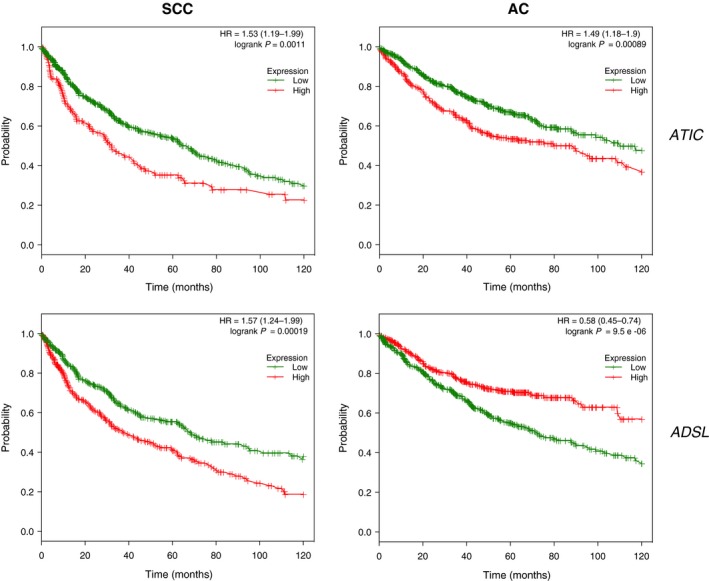
Association between *ADSL/ATIC* genetic expression and survival in LC cohorts. Kaplan–Meier plots for *ADSL* and *ATIC* genetic expression (high and low levels) in LC cohorts available from kmplotter (*n* = 724 for AC and *n* = 524 for SCC). Log‐rank *P*‐values and hazard ratios (HR; 95% confidence interval in parentheses) are shown. The *P*‐value represents the equality of survival curves based on a log‐rank test.

Finally, we decided to study, in both histological subtypes, the most relevant enzymes involved in the regulation of purine catabolism, selecting the top 10 according to their role in the pathway (Fig.  S4). As shown in Fig. 7A, we found significant changes in the genetic expression of six enzymes, four of which presented different expression patterns in both histological subtypes. Due to the importance of purinosome as a possible new therapeutic target in the treatment of LC (Jackson *et al*., 2013), we decided to look closely into the analysis of ATIC and ADSL. First, analyses of available data from The Cancer Genome Atlas (TCGA)  (*n* = 230 for AC and *n* = 179 for SCC) revealed that 7% of the human SCC cases and 9% of the AC cases present genetic alterations in *ATIC*, which are associated with deep deletion and mRNA downregulation in SCC, and amplification and mRNA upregulation in AC. Similarly, in the case of *ADSL,* 18% of the human SCC cases and 8% of the AC cases present genetic alterations, generally mRNA upregulation (Fig. 7B). These results are similar to those obtained with our series of data. The analysis of ATIC and ADSL protein expression through immunohistochemistry (Fig. 7C,D) showed much more intense expression in tumor than in healthy tissue for AC and SCC cases. Finally, we analyzed *ATIC* or *ADSL* expression with the information of general and relapse‐free survival in both histological subtypes available from TCGA and several Gene Expression Omnibus studies (*n* = 724 for AC and *n* = 524 for SCC). As shown in Fig. 8, in all cases there is a clear significant relation between survival and *ATIC* or *ADSL* expression. Interestingly, in the case of *ADSL* expression, there are opposing associations with survival depending on the histological subtypes analyzed.

## 4 Discussion

This study represents, to our knowledge, the widest research to date performed in lung tissue to find the metabolic differences between tumors of the most important histological subtypes and normal tissue from the same patient. We have mainly selected tumors at an early stage, so that we could keep track of the initial biochemical perturbations taking place in the process of tumorigenesis.

Detailed analysis of the glucose metabolism showed changes consistent with a Warburg effect, a signature of cancer metabolism where rapidly dividing cells display an elevated glucose uptake and an activated glycolysis with an increased activity of the pentose phosphate pathway as required for the production of nucleotides (Fig. 2) (Jiang *et al*., 2014). Further support of this includes the accumulation of tricarboxylic acid cycle intermediates, together with a significant elevation of pentose sugar alcohols and amino sugars. Furthermore, higher levels of sorbitol can contribute to the generation of glycation products as reported in the LC literature (Ahmad *et al*., 2018), which were also detected in the present study. Higher lactate levels may result from the rapid shuttling of these glycolytic metabolites to support energy generation. Together, these findings suggest alterations in glucose uptake and utilization, which may support an increase in pentose phosphate pathway activity and the generation of glycation products that may potentially contribute to the pathogenesis and growth of these tumors.

Related to the glutathione levels and additional markers of oxidative stress, the increase observed in MetO, reduced glutathione, cysteine and glycine levels in both tumor subtypes provides further support for changes associated with an increase in oxidative stress (Fig. 3A). In this sense, cysteine has been shown to be elevated in various types of cancer including LC (Gamcsik *et al*., 2012; Krepela *et al*., 1997), and the glycine decarboxylase, the enzyme responsible for glycine degradation, has been related to tumor‐initiating cells in NSCLC (Berezowska *et al*., 2017; Lin *et al*., 2017). Interestingly, there were also consistently elevated levels of gamma‐glutamyl amino acids, which are generated by gamma‐glutamyl transpeptidase. Altered gamma‐glutamyl transpeptidase levels are a clear marker of oxidative stress and are often significantly increased in human malignancy (Corti *et al*., 2010). Finally, the high levels of powerful antioxidants detected, such as ascorbate and alpha/gamma‐tocopherol, support the idea that an accumulation of oxidative stress markers and antioxidants takes place in lung tumors.

Regarding arginine metabolism, we observed a slight difference in arginine ornithine and citrulline, as well as in aspartate levels (Fig. 3B). Initially, these results were unexpected, since previous studies reported an increase of these metabolites in some types of cancer (Wheatley and Campbell, 2002), and ornithine decarboxylase, the enzyme responsible for putrescine biogenesis, is significantly elevated in multiple LCs (Grimminger *et al*., 2010; Tian *et al*., 2006). However, a previous metabolomic study in AC showed similar results, which suggests the need for a detailed study of this aspect (Wikoff *et al*., 2015). In contrast, higher levels in tumor tissues of other polyamines (putrescine, spermine and spermidine), as well as related molecules (*N*‐acetylputrescine and MTA), critical regulators of nucleic acid stabilization and cell cycle progression, were detected (Gerner and Meyskens, 2004; Pegg, 2009). These results, along with the higher levels detected of proline, Pro‐hydroxy‐Pro or dimethylarginine in the tumor samples, suggest an increase in extracellular matrix remodeling, cellular proliferation and lung function (Rafikova *et al*., 2016; Wells *et al*., 2009).

These alterations are consistent with the changes observed in the metabolism of fatty acids and lipid mediators. Cancerous lung tissue exhibited a significant accumulation of multiple long‐chain fatty acids, as well as monoacylglycerols and glycerol (Fig. 4A), which is indicative of increased or altered beta‐oxidation (Nomura *et al*., 2010). Differences in phospholipid metabolism can reflect the growth and turnover of tissue as well as the increase after degradation of membranes during apoptosis (Beloribi‐Djefaflia *et al*., 2016; Santos and Schulze, 2012). Notably, citrate, carnitine and carnitine‐conjugated lipids were significantly lower in LC tissue. Decreased levels of citrate may suggest a limited availability of this metabolite to support lipid synthesis, and reductions in carnitine conjugate lipids may reflect a decrease in lipid transport capacity into the mitochondria (Currie *et al*., 2013). In summary, these changes support membrane growth and cellular proliferation, which is consistent with the changes in polyamine levels observed.

Another pathway of intense interest in cancer in recent years is one‐carbon metabolism, mainly due to the need of cancer cells to support enhanced proliferation and survival (Ducker and Rabinowitz, 2017; Newman and Maddocks, 2017). In this study, multiple pathway metabolites were significantly altered (glycine, sarcosine, SAH, methionine, homocysteine; Fig. 4B). Previous literature shows that to sustain a one‐carbon supply for proliferation, cancer cells convert serine to glycine, generating methylene‐5‐methyltetrahydrofolate (Tibbetts and Appling, 2010). Moreover, it has been suggested that glycine promotes cancer cell growth in lung tumor‐initiating cells (Zhang *et al*., 2012). The significant accumulation of sarcosine may result in increased biogenesis, as supported by SAH, and has been shown to be correlated with increased methylation and tumor aggressiveness in other cancers (Cernei *et al*., 2013).

Finally, and as discussed above, we observed a significant increase in ribose‐5‐phosphate levels in tumor tissue. Recently, it has been described that tumor cells redirect glucose flow to generate ribose‐5‐phosphate in order to increase the synthesis of nucleotides and thus maintain a high rate of growth (Ciou *et al*., 2015). These data, together with the relevant role of nucleotide catabolism for DNA replication, led us to analyze this pathway in detail. First, higher levels of initial products such as AMP were detected in tumor tissues, which were accompanied by the absence of significant changes in GMP (Fig. 5A). These data suggest a shift in the pathway favoring AMP synthesis. The analysis of the differential expression of the enzymes involved in this pathway showed a significant increase in GMPR and ADSL expression in SCC and AC as compared with normal tissue (Fig. 7A). The enzyme GMPR catalyzes a reverse reaction which turns GMP into IMP, keeping these metabolite levels high. Supporting these results, we also found an overexpression of ADSL, the enzyme in charge of AMP synthesis, both at a genetic and at a protein level in tumor tissue for AC and SCC cases (Fig. 7). These data prove the existence of an activation of the *de novo* synthesis favoring AMP synthesis, which reflects enhanced ATP/AMP turnover as supported by significantly higher levels of multiple purine catabolic products such as inosine, hypoxanthine, xanthine and xanthosine.

In relation to these results, the ADSL enzyme has been described as relevant to the formation of a complex called purinosome, formed by six enzymes in charge of performing 10 sequential reactions in the step from phosphoribosyl pyrophosphate to IMP (An *et al*., 2008). This complex is formed under situations of high purine demand in order to increase the metabolic flow of the *de novo* synthesis and complement the recovery path. Although in some respects the existence of this complex can be discussed, the alterations in the *de novo* pathway in several human diseases has caused the purinosome to be considered a therapeutic target for clinical treatments (Zhao *et al*., 2013), including in oncology (Zhang *et al*., 2015). In this sense, other groups have described the deficiency of ADSL and ATIC as affecting the formation and assembly of purinosome, and there is evidence that both enzymes are key in the regulation of the synthesis pathway of purines during tumor development (Baresova *et al*., 2012; Jurecka *et al*., 2015). Our results prove the existence of an increase in the expression of both enzymes at the protein level in lung tumor tissue compared with healthy tissue from the same patient in SCC and AC. These results are also validated by analysis of available data from public cancer genomics studies. Furthermore, we show the existence of a clear significant relation between survival and *ATIC* or *ADSL* expression (Fig. 8). The opposite association with survival observed in the expression of *ADSL* depending on the histological subtypes analyzed is especially relevant, since it could be due to a gene with a key role in the differentiation between both histological subtypes. All these data confirm the potential role of purinosome as a therapeutic treatment for LC.

We also detected an increase in the expression of metabolites involved in the pyrimidine pathway, such as CMP, uridine, cytidine, uracil and 5,6‐dihydrouracil (Fig. 5A), in tumors. In this sense, dihydropyrimidine dehydrogenase, the enzyme responsible for the production of 5,6‐dihydrouracil as the uracil oxidation product in humans, shows a higher activity and expression in lung AC than in control tissue. Moreover, its activity has also been related to improved efficacy of cytotoxic effects from common postoperative adjuvant therapy NSCLC (Miyoshi *et al*., 2005; Shintani *et al*., 2011). Recently, 5,6‐dihydrouracil has been shown to be elevated in AC (Wikoff *et al*., 2015).

Finally, the statistical analysis of the results of the present study is evidence of the existence of changes in the nucleotide metabolism in LC tissue vs. normal tissue, which were in particular more relevant in SCC rather than in AC. These results also revealed the existence of differences in the levels of metabolites in AC vs. SCC cases, which means that both histological subtypes regulate the nucleotide metabolism in a different manner (Fig. 5B). This fact was supported by the comparison of the metabolites with the highest discrimination capability for AC and SCC (Fig. 5C). In the case of AC, the predictive capability of the top five metabolites analyzed was highly relevant. Sensitivity was always above 0.787 and specificity above 0.818, especially noted for 5,6‐dihydrouracil (Fig. 6A). The predictive capability was even better for the SCC subtype; sensitivity and specificity values were always above 0.914, with special emphasis on 2′‐O‐methylguanosine and 5‐methyluridine, the two metabolites with the highest discriminatory capacity. In fact, there was practically no overlap in SCC tissue and the corresponding normal tissue seen in the box plots of the concentration range detected for both metabolites in lung tissue (Fig. 6B). A trend found for nucleotides was that their concentrations were characterized by a common pattern. They were always more concentrated in the cancerous tissues than in normal tissues, both for AC and SCC cases, which is clearly indicative of hypermetabolism.

## 5 Conclusions

This work provides a detailed analysis of the metabolomic changes taking place in relevant biochemical pathways of the most important histological subtypes of LC, with a special interest in nucleotide metabolism. Additionally, the obtained results allow identification of the potential discriminatory role of metabolites for the two most diagnosed lung carcinoma types and, therefore, their possible predictive capability and their use as diagnostic markers. Detailed knowledge of these alterations also opens the door for the discovery of new therapeutic intervention targets.

## Authors’ contributions

PM, CJJ, MGR, MCS, SM and MLC designed and performed the experiments and analyzed the data; FPC, AS and EM contributed conceptual input; MAC conceived the study, analyzed the data, wrote the manuscript and approved the final version to be published; and all authors read and approved the final manuscript.

## Supporting information


**Fig. S1**. Unsupervised analysis by PCA applied to the complete dataset.

**Fig. S2**. Unsupervised analysis by PCA applied to the nucleotide metabolic pathway for AC and SCC vs. normal tissue.

**Fig. S3**. PLS‐DA models.

**Fig. S4**. Enzymes involved in the regulation of purine catabolism.

**Data S1**. Dataset with completetly different metabolic profiles obtained for AC and SCC (excel file).
