# Supplementary material for: Metabolomic profiling of human lung tumor tissues – nucleotide metabolism as a candidate for therapeutic interventions and biomarkers
Source: Mol Oncol. 2018 Sep 13;12(10):1778–96. doi: 10.1002/1878-0261.12369 (PMC6165994; doi:10.1002/1878-0261.12369)
Supplement: Supplementary file 1 — Fig. S1. Unsupervised analysis by PCA applied to the complete dataset. [file MOL2-12-1778-s001.pdf]

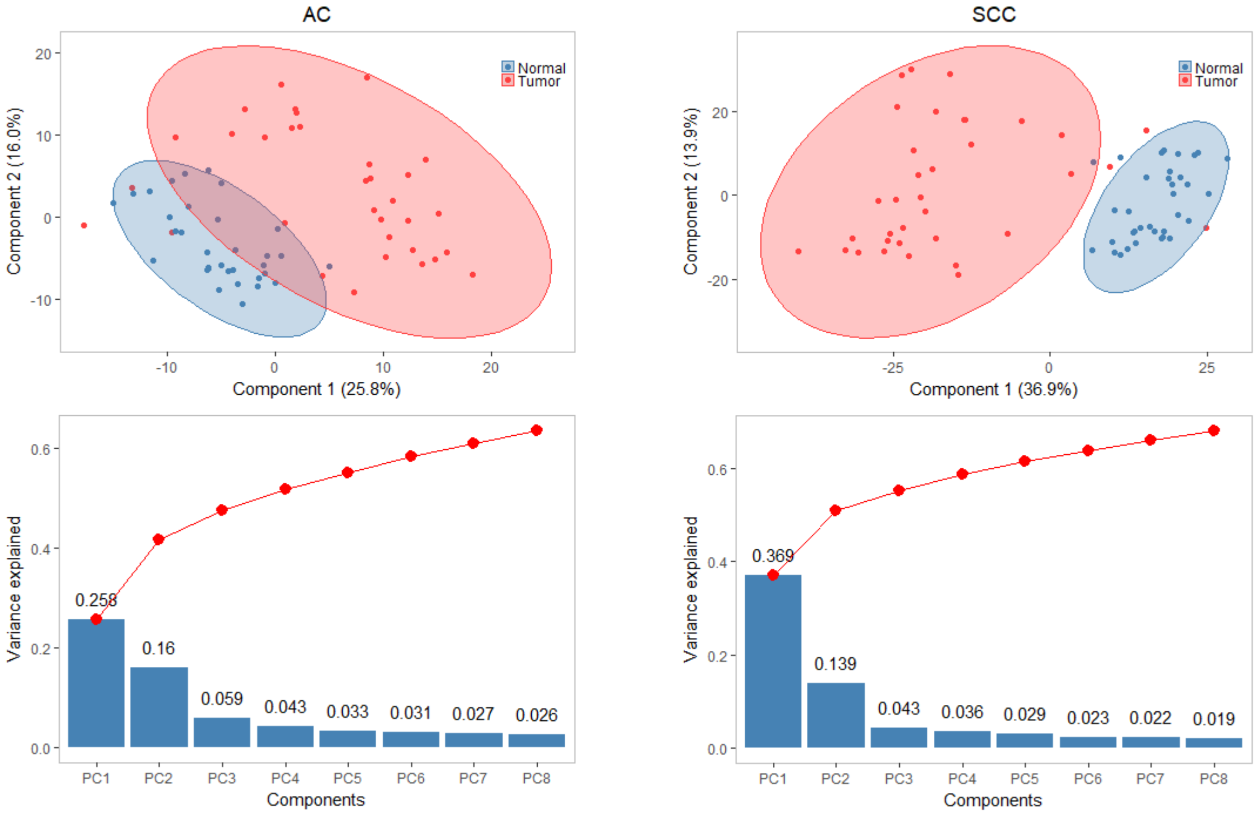


**Fig. S1. Unsupervised analysis by PCA applied to the complete dataset.** PCA plots provided by the complete data set that show discrimination patterns between adenocarcinoma (AC) lung tissue versus control tissue and squamous lung carcinoma tissue (SCC) versus control tissue. The contribution of the principal components (PC) to explain the observed variability and the cumulative contribution are also illustrated.
