# Supplementary material for: Metabolomic profiling of human lung tumor tissues – nucleotide metabolism as a candidate for therapeutic interventions and biomarkers
Source: Mol Oncol. 2018 Sep 13;12(10):1778–96. doi: 10.1002/1878-0261.12369 (PMC6165994; doi:10.1002/1878-0261.12369)
Supplement: Supplementary file 2 — Fig. S2. Unsupervised analysis by PCA applied to the nucleotide metabolic pathway for AC and SCC vs. normal tissue. [file MOL2-12-1778-s002.pdf]

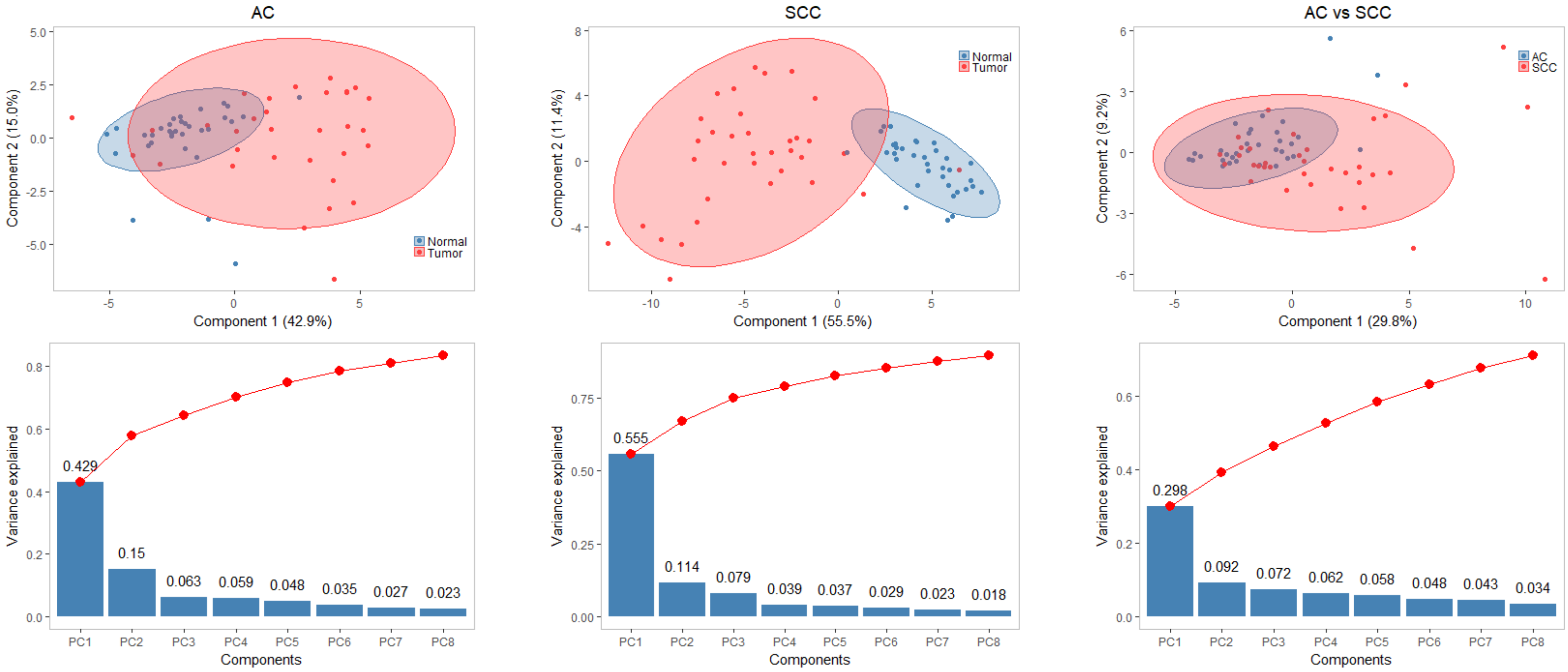

Fig. S2. Unsupervised analysis by PCA applied to the nucleotide metabolic pathway for AC and SCC versus normal tissue. PCA plots provided by purines data set that show discrimination patterns between adenocarcinoma (AC) lung tissue versus control tissue, squamous lung carcinoma tissue (SCC) versus control tissue, and AC versus SCC lung tissues, the latter obtained considering the fold change value of each metabolite between normal and cancerous tissue in order to compare both types of carcinomas. The contribution of the principal components (PC) to explain the observed variability and the cumulative contribution are also illustrated.
