# Supplementary material for: Metabolomic profiling of human lung tumor tissues – nucleotide metabolism as a candidate for therapeutic interventions and biomarkers
Source: Mol Oncol. 2018 Sep 13;12(10):1778–96. doi: 10.1002/1878-0261.12369 (PMC6165994; doi:10.1002/1878-0261.12369)
Supplement: Supplementary file 3 — Fig. S3. PLS‐DA models. [file MOL2-12-1778-s003.pdf]

Figure S3

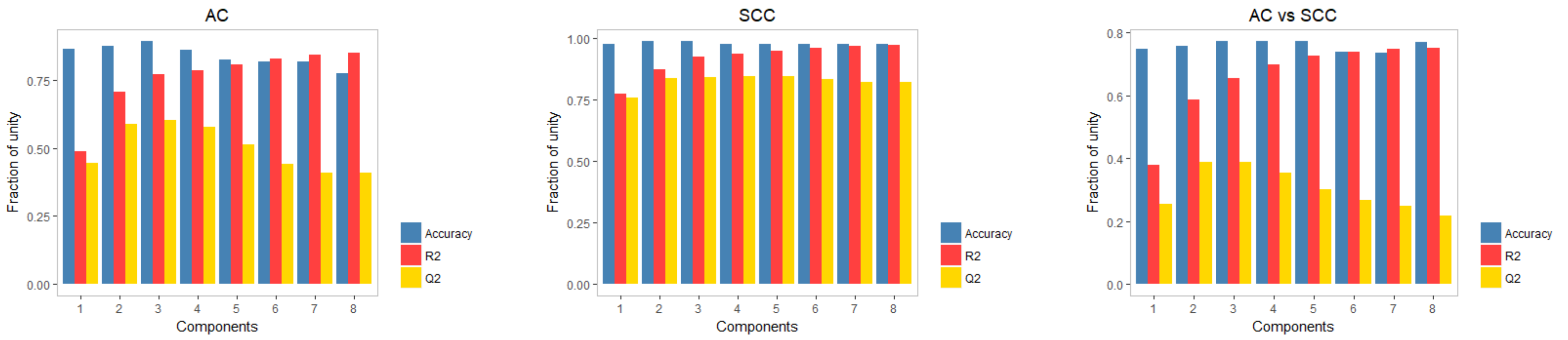

Fig. S3. PLS-DA models. Performance indicators expressed in terms of accuracy, goodness of fit (R^2) and prediction (Q^2) for combination of components for the three PLS-DA models discriminating adenocarcinoma (AC) lung tissue versus control tissue, squamous lung carcinoma tissue (SCC) versus control tissue, and AC versus SCC lung tissues.
