# Supplementary material for: Metabolomic profiling of human lung tumor tissues – nucleotide metabolism as a candidate for therapeutic interventions and biomarkers
Source: Mol Oncol. 2018 Sep 13;12(10):1778–96. doi: 10.1002/1878-0261.12369 (PMC6165994; doi:10.1002/1878-0261.12369)
Supplement: Supplementary file 4 — Fig. S4. Enzymes involved in the regulation of purine catabolism. [file MOL2-12-1778-s004.pdf]

Symbol	Name
ADA	Adenosine deaminase
IMPDH2	Inosine-5'-monophosphate dehydrogenase 2
GMPS	Guanosine monophosphate synthetase
GMPR	Guanosine Monophosphate Reductase
XDH	Xanthine dehydrogenase
ATIC	IMP cyclohydrolase
HPRT1	Hypoxanthine Phosphoribosyltransferase 1
ITPA	Inosine triphosphate pyrophosphatase
ADSL	Adenylosuccinate lyase
ADSS	Adenylosuccinate synthase

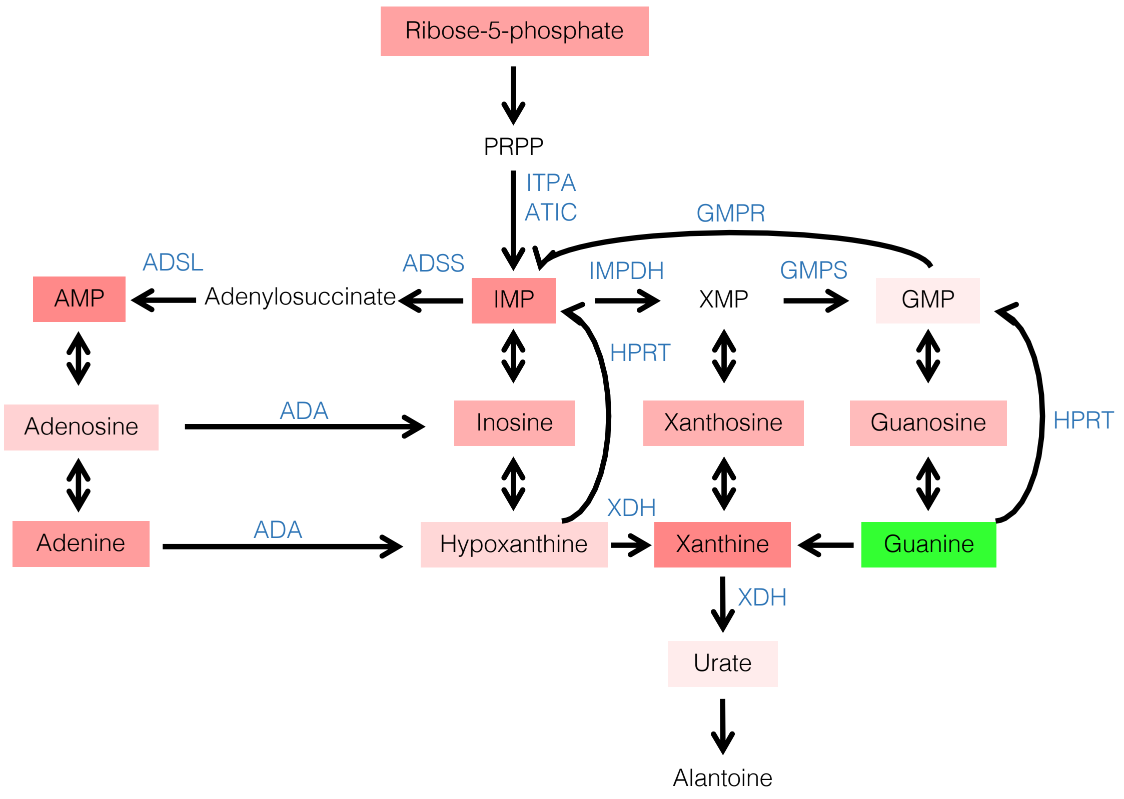

Fig. S4. Enzymes involved in the regulation of purine catabolism. Enzymes analyzed involved in the Nucleotide metabolism pathway.
