# Supplementary material for: Metabolomic profiling of human lung tumor tissues – nucleotide metabolism as a candidate for therapeutic interventions and biomarkers
Source: Mol Oncol. 2018 Sep 13;12(10):1778–96. doi: 10.1002/1878-0261.12369 (PMC6165994; doi:10.1002/1878-0261.12369)
Supplement: Supplementary file 6 [file MOL2-12-1778-s006.docx]

**Supplementary information for:**

**Metabolomic profiling of human lung tumor tissues: nucleotide metabolism as a candidate for therapeutic interventions and biomarkers.**

Paula Moreno^1,3*^, Carla Jiménez-Jiménez^1,2*^, Martín Garrido^5^, Mónica Calderón-Santiago^1,4^, Susana Molina^1,2^, Maribel Lara-Chica^1,2^, Feliciano Priego-Capote^1,4^, Ángel Salvatierra^1,3^, Eduardo Muñoz^1,2^ and Marco A. Calzado^1,2^

^1^ Instituto Maimónides de Investigación Biomédica de Córdoba (IMIBIC), Córdoba, Spain.

^2^ Departamento de Biología Celular, Fisiología e Inmunología, Universidad de Córdoba, Córdoba, Spain.

^3^ Unidad de Cirugía Torácica y Trasplante Pulmonar. Hospital Universitario Reina Sofía, Córdoba, Spain.

^4^ Departamento de Química Analítica, Universidad de Córdoba, Córdoba, Spain.

^5^ Innohealth Group, Madrid, Spain.

*These authors contributed equally to this study.

**Corresponding Author:** Marco A. Calzado Ph.D.

Instituto Maimónides de Investigación Biomédica de Córdoba (IMIBIC)

Avda. Menendez Pidal s/n. 14004, Córdoba, Spain

Phone: + 34 957213762

e-mail: [mcalzado@uco.es](mailto:mcalzado@uco.es)

**Supplementary Table Legends**

**Supplementary Data 1.** Data set with total different metabolic profiles obtained for AC and SSC. (Excel file)

**Supplementary Figure Legends**

**Figure S1.** PCA plots provided by the complete data set that show discrimination patterns between adenocarcinoma (AC) lung tissue versus control tissue and squamous lung carcinoma tissue (SCC) versus control tissue. The contribution of the principal components (PC) to explain the observed variability and the cumulative contribution are also illustrated.

**Figure S2.** PCA plots provided by purines data set that show discrimination patterns between adenocarcinoma (AC) lung tissue versus control tissue, squamous lung carcinoma tissue (SCC) versus control tissue, and AC versus SCC lung tissues, the latter obtained considering the fold change value of each metabolite between normal and cancerous tissue in order to compare both types of carcinomes. The contribution of the principal components (PC) to explain the observed variability and the cumulative contribution are also illustrated.

**Figure S3.** Performance indicators expressed in terms of accuracy, goodness of fit (*R^2^*) and prediction (*Q^2^*) for combination of components for the three PLS-DA models discriminating adenocarcinoma (AC) lung tissue versus control tissue, squamous lung carcinoma tissue (SCC) versus control tissue, and AC versus SCC lung tissues.

**Figure S4.** Enzymes analyzed involved in the Nucleotide metabolism pathway.
